# First person – Priyanka Tibarewal

**DOI:** 10.1242/dmm.052724

**Published:** 2026-01-07

## Abstract

First Person is a series of interviews with the first authors of a selection of papers published in Disease Models & Mechanisms, helping researchers promote themselves alongside their papers. Priyanka Tibarewal is first author on ‘ Impaired nuclear PTEN function drives macrocephaly, lymphadenopathy and late-onset cancer in PTEN hamartoma tumour syndrome’, published in DMM. Priyanka is a senior research associate in the lab of Bart Vanhaesebroeck at University College London, London, UK, investigating the disease mechanism and repurposing drugs for PTEN hamartoma tumour syndrome (PHTS).


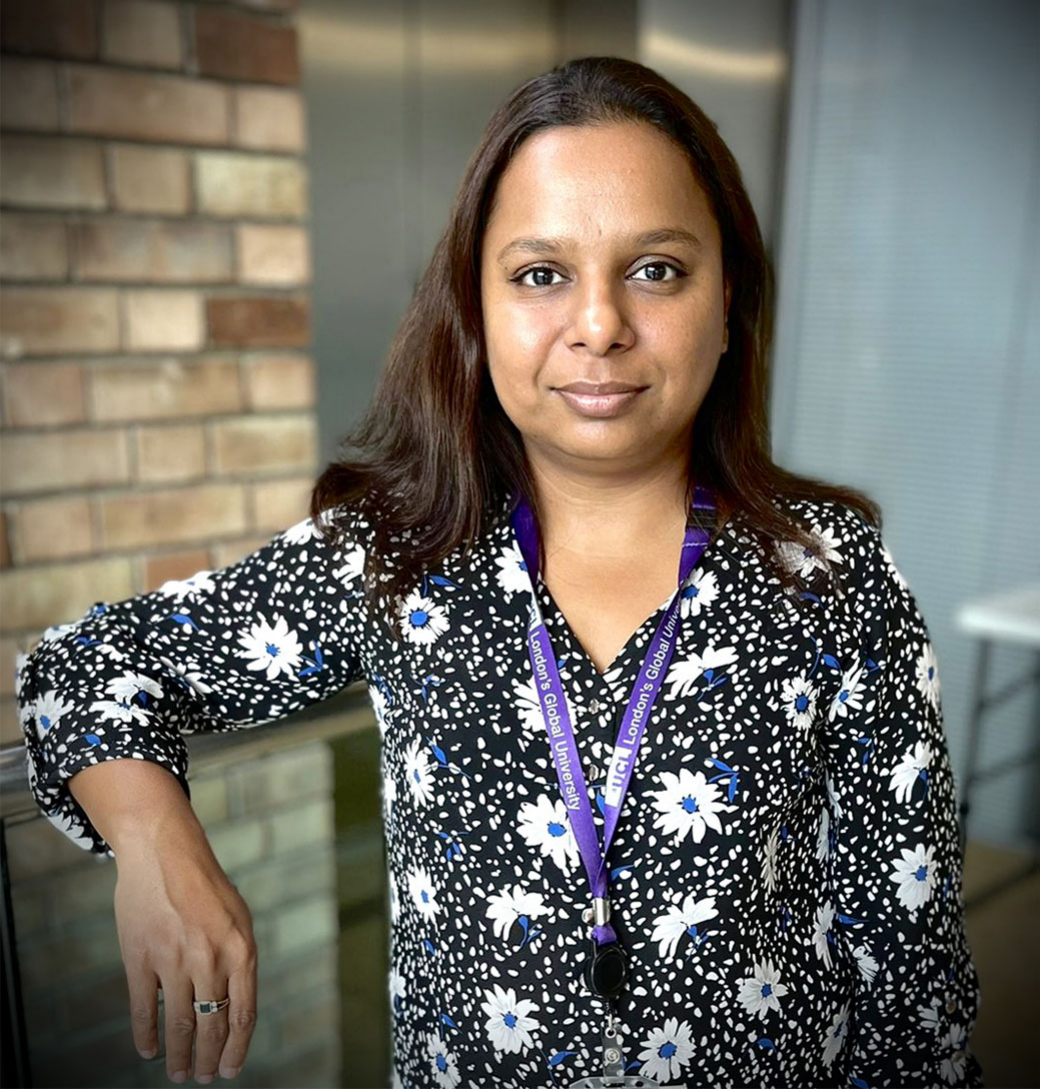


**Priyanka Tibarewal**

**Who or what inspired you to become a scientist?**

I've always loved biology. Something about how living things work fascinated me. That curiosity led me to study biological sciences at university. During my studies, I worked in hospitals and research labs, learning about human diseases. I enjoyed asking questions and finding answers through research. It felt exciting and meaningful. Around that time, my grandmother was diagnosed with breast cancer. Her illness made me want to understand cancer better and help others facing it. That personal experience pushed me toward cancer research. I saw how science could make a real difference in people's lives. That's what inspired me to become a scientist.

**What is the main question or challenge in disease biology you are addressing in this paper? How did you go about investigating your question or challenge?**

PHTS is a rare genetic condition caused by mutations in the *PTEN* tumour suppressor gene. People with PHTS have a higher risk of developing cancers in different tissues. They can also show a wide range of other symptoms, including a large head (macrocephaly) because of brain overgrowth, developmental delays, autism spectrum disorder, skin overgrowths, vascular issues and immune problems. One major challenge is that symptoms vary a lot between patients, even between siblings with the same mutation.

In our paper, we aim to understand what causes this variability. Because PHTS is rare, there isn't much patient data available, and research often depends on using mouse models. We faithfully modelled the genetic constellation seen in PHTS patients in mice, and combine insights from both patient data and mouse studies to explore how *PTEN* mutations affect different biological pathways and if that explains range of outcomes seen in PHTS. We hope our findings can improve patient diagnosis, care and treatment.

**How would you explain the main findings of your paper to non-scientific family and friends?**

In our study, we explored how different roles of the *PTEN* gene might explain the wide range of health issues seen in people with PHTS. By combining insights from lab research in mice with real-world data from patients, we found that certain changes in PTEN, specifically those affecting its work inside the cell's control centre (the nucleus), are linked to larger head size and developmental delay during childhood. These individuals often develop harmless growths in early adulthood and, later in life (usually after age 60), face a higher risk of cancer. Our findings aim to inform surveillance strategies and improve lifelong care for individuals living with PHTS.

… we found that certain changes in PTEN, specifically those affecting its work inside the cell's control centre (the nucleus), are linked to larger head size and developmental delay during childhood

**What are the potential implications of these results for disease biology and the possible impact on patients?**

The findings highlight critical implications for both disease biology and patient care in PHTS. The observed variability in symptom type and age of onset suggests that PHTS may encompass distinct subgroups, potentially driven by specific *PTEN* variant characteristics, such as PTEN no longer being present in the cell nucleus. This insight deepens our understanding of genotype–phenotype relationships and the temporal dynamics of disease progression.

For patients, these results underscore the need to move beyond a one-size-fits-all approach for diagnosis and care. Identifying molecular drivers of heterogeneity could pave the way for personalised surveillance and treatment strategies and, ultimately, could reduce morbidity and improve quality of life for those most vulnerable to early symptom manifestations.

**Figure:**
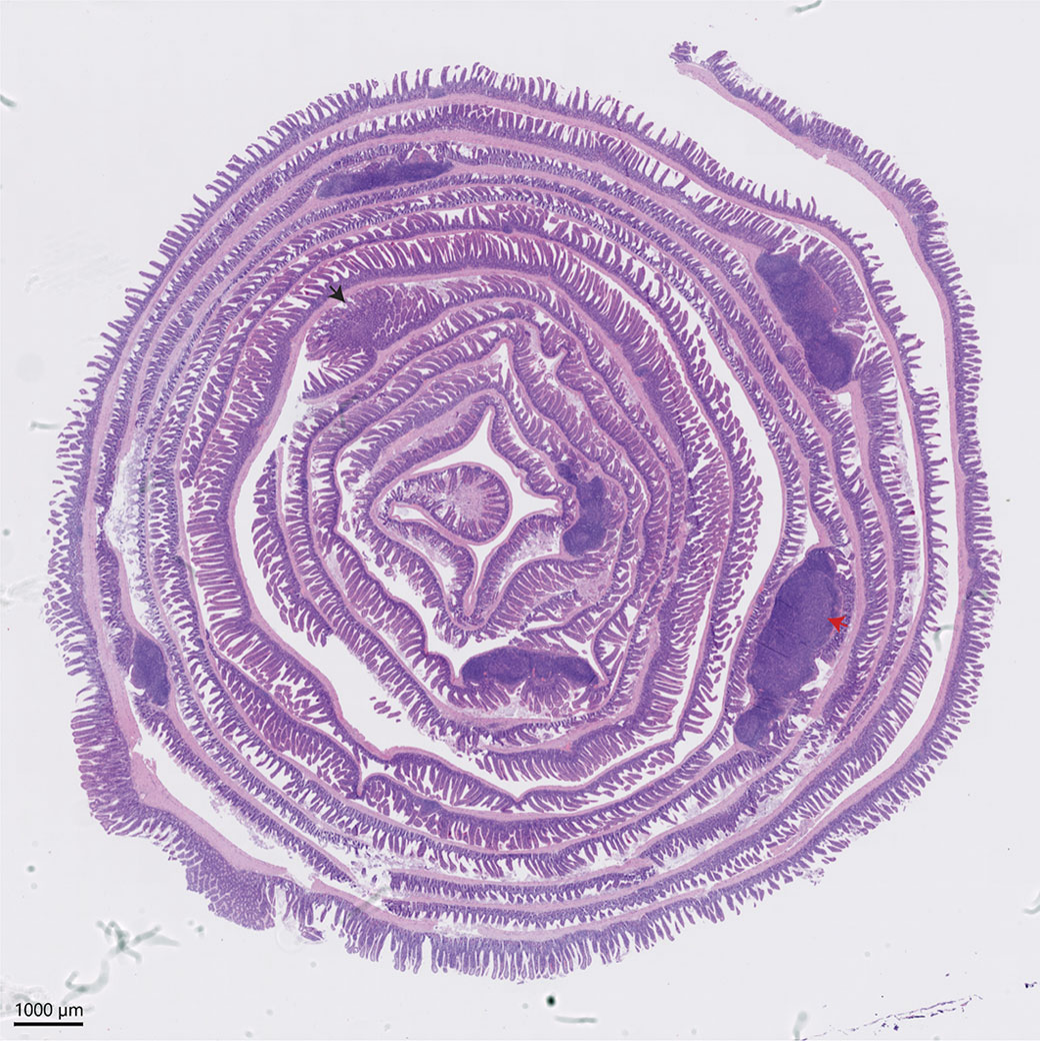
**A mouse gastrointestinal tract, nearly 50 cm long, rolled into a ‘Swiss roll’ to fit on a single histological slide to allow viewing it in its entirety.** This image highlights hyperplastic glands (black arrow) and gut-associated lymphoid tissue (red arrow).

**Why did you choose DMM for your paper?**

We chose DMM for our paper because its scope and mission closely align with our work and ambitions. Our study models the rare genetic disorder PHTS and investigates the underlying mechanisms driving a subset of its manifestations, specifically cancer, neurological and immunological symptoms. DMM's emphasis on translational research and its commitment to advancing understanding of disease biology through model systems make it an ideal platform for sharing our findings with both basic and clinical research communities.

**Given your current role, what challenges do you face and what changes could improve the professional lives of other scientists in this role?**

As a rare disease researcher, I face several challenges that impact both the progress of science and the wellbeing of those affected by these conditions. One of the most pressing issues is the lack of dedicated funding. Rare disease research often relies on small charities or niche funding bodies, which limits the scale, continuity and innovation of scientific efforts. Another significant challenge is the limited number of publication platforms that prioritise rare disease research. Without accessible and reputable outlets to share findings, important insights risk being overlooked or delayed in reaching clinicians, patients and fellow researchers.

To improve the professional lives of scientists in this space, we need more robust and sustained funding mechanisms, ideally through government agencies, philanthropic foundations and industry partnerships. Additionally, expanding journal platforms and creating dedicated spaces for rare disease research would foster visibility, collaboration and impact. These changes would empower researchers to pursue bold questions and translate discoveries into meaningful outcomes for underserved patient communities.

… expanding journal platforms and creating dedicated spaces for rare disease research would foster visibility, collaboration and impact

**What's next for you?**

PHTS is a rare and complex condition. As researchers, we've made important strides in understanding how it works at a biological level, but it's easy to lose sight of what patients actually go through. I've had the privilege of connecting with the PHTS community in the UK through PTENUKI, a research charity for which I also serve as a trustee. These conversations have deeply shaped my perspective. I now better understand how PHTS affects people's daily lives and the many challenges they face, including the lack of approved treatments.

To help address this, my current research focuses on testing existing drugs to see if they could be repurposed for PHTS. These early-stage studies aim to lay the foundation for future therapies. Ultimately, my goal is to answer the kinds of questions that can improve care and bring us closer to safe, personalised treatment options for PHTS patients.

**Tell us something interesting about yourself that wouldn't be on your CV**

Something interesting about me that wouldn't appear on my CV is my love for baking. For me, it's more than just a hobby, it is almost like a science experiment in the kitchen, just with a sweeter outcome.
